# Versatile on-chip polarization-sensitive detection system for optical communication and artificial vision

**DOI:** 10.1038/s41377-025-01744-x

**Published:** 2025-02-03

**Authors:** Zhilin Liu, Mingxiu Liu, Liujian Qi, Nan Zhang, Bin Wang, Xiaojuan Sun, Rongjun Zhang, Dabing Li, Shaojuan Li

**Affiliations:** 1 Key Laboratory of Luminescence Science and Technology, Chinese Academy of Sciences & State Key Laboratory of Luminescence Science and Applications, Changchun Institute of Optics, Fine Mechanics and Physics, Chinese Academy of Sciences, Changchun, Jilin 130033 China; 2 University of Chinese Academy of Sciences, Beijing, 100049 China; 3 Department of Optical Science and Engineering, Shanghai Frontiers Science Research Base of Intelligent Optoelectronics and Proception, Institute of Optoelectronics, Fudan University, Shanghai, 200433 China

**Keywords:** Optoelectronic devices and components, Imaging and sensing

## Abstract

Polarization is an important attribute of light and can be artificially modulated as a versatile information carrier. Conventional polarization-sensitive photodetection relies on a combination of polarizing optical elements and standard photodetectors, which requires a substantial amount of space and manufacturing expenses. Although on-chip polarized photodetectors have been realized in recent years based on two-dimensional (2D) materials with low-symmetry crystal structures, they are limited by the intrinsic anisotropic property and thus the optional range of materials, the operation wavelength, and more importantly, the low anisotropic ratio, hindering their practical applications. In this work, we construct a versatile platform that transcends the constraints of material anisotropy, by integrating WSe_2_-based photodetector with MoS_2_-based field-effect transistor, delivering high-performance broadband polarization detection capability with orders of magnitude improvement in anisotropic ratio and on/off ratio. The polarization arises from hot electron injection caused by the plasmonic metal electrode and is amplified by the transistor to raise the anisotropic ratio from 2 to an impressive value over 60 in the infrared (IR) band, reaching the level of existing applications. Meanwhile, the system achieves a significant improvement in photosensitivity, with an on/off ratio of over 10^3^ in the IR band. Based on the above performance optimization, we demonstrated its polarization-modulated IR optical communication ability and polarized artificial vision applications with a high image recognition accuracy of ~99%. The proposed platform provides a promising route for the development of the long-sought minimized, high-performance, multifunctional optoelectronic systems.

## Abstract (web summary)

A versatile polarization detection platform that surpasses the limitation of material anisotropy is demonstrated for optical communication and artificial vision with a high polarization ratio.

## Graphical abstract

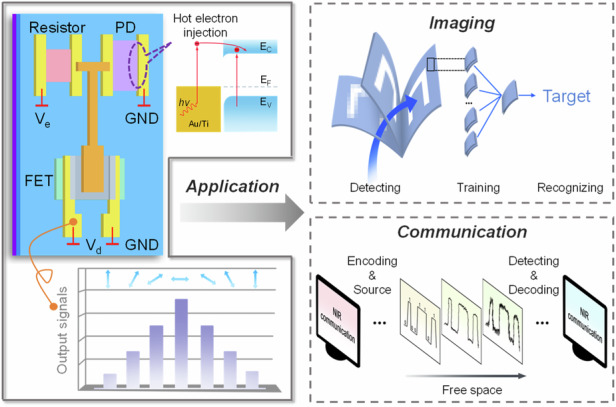

## Introduction

**Fig. 1:**
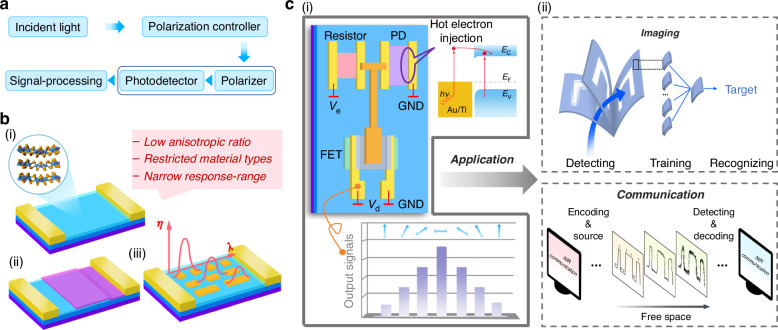
The working principle and application schematics of the proposed ASPD system. **a** Traditional polarized detection process illustrated in a block diagram. **b** Overview of the existing 2D materials-based polarized photodetectors and their limitations. These include: (i) Anisotropic 2D materials. The inset shows a typical schematic structure of the anisotropic 2D material. (ii) Heterojunction construction. (iii) Coupling with anisotropic nanostructures. This method introduces polarization through anisotropic absorption or field enhancement via localized surface plasmon resonance (LSPR) of nanostructures. The red coordinate illustrates the relationship between resonance wavelength, denoted by *λ*, and processing difficulty, denoted by *η*. The fixed size of nanostructures features a specific resonance wavelength with a finite spectral width, with broader wavelengths correlating to increased processing complexity. **c** (i) Schematic of the ASPD system integrating polarization-sensitive WSe_2_-based photodetector and MoS_2_-based field-effect transistor. In this schematic, PD denotes a WSe_2_-based photodetector, FET denotes a MoS_2_-based transistor, and Resistor denotes a reference resistor. The right top panel indicates that polarization sensitivity arises from hot electron injection. The bottom panel depicts the output signals of the ASPD system that vary with the incident polarization angle. (ii) Application schematics of the ASPD system in imaging and optical communication, showcasing its versatility

Multifunctional photodetectors (PDs) that convert multiple attributes of light signals into electrical readouts are one of the key components of modern optoelectronic technologies^1–6^. Among them, polarization-sensitive PDs can effectively detect polarization state and intensity of light, enhance the information dimensions and imaging contrast of targets^7^, playing a crucial role in the fields of optical communication^8^, spectroscopy^9–11^, remote polarization imaging^12,13^, and artificial vision^14,15^. Recently, the requirements of the next-generation miniaturized on-chip optoelectronic system have pushed the efforts to search for highly integrated polarization detection technology^5,16–18^. However, traditional polarization photodetection systems that relies on a combination of polarizing optical elements and conventional PDs (Fig. 1a), are limited by the bulky volume, complicated fabrication and high cost^19,20^.

Two-dimensional (2D) materials have shown tremendous potential to solve the above limitations with their excellent optoelectronic and physical properties^21–23^. In particular, 2D materials with low-symmetry crystal structures, such as BP^24^, ReS_2_^25^, PdSe_2_^26^, Ta_2_NiSe_5_^27^, etc., which can realize polarization detection without polarizers (Fig. 1b(i)), have been verified and opened a new pathway for the development of on-chip polarization-sensitive photodetection. However, so far most polarization-sensitive PDs based on these low-symmetry 2D materials still face the challenges of limited responsivity, low signal-to-noise ratio, and unsatisfying anisotropic ratio (normally <10)^28^, which hampers their further development of applications. Besides, the variety of low-symmetry crystal 2D materials, of which there are only a few dozens^29^, is quite limited compared to the vast family of 2D materials (~6300)^30^. Many 2D materials with superior optoelectronic properties cannot be directly applied to polarized PDs, such as WSe_2_^31^, and MoS_2_^32^. Although some existing strategies, such as building heterostructures (Fig. 1b(ii))^33,34^ and coupling artificial nanostructures (Fig. 1b(iii))^35,36^, may compensate for this deficiency, these methods are not universally applicable and face challenges in terms of preparation techniques, large-area integration, processing complexity and so on.

Over the last decades, developments in plasmonic have provided an efficient route for enhancing light-matter interactions, localized field enhancement, and resonant absorption^37–40^. Hot carriers generated by the non-radiative decay of plasmon excitations can be extracted by electron acceptors such as metal-semiconductor Schottky interfaces or metal-insulator-metal junctions^41,42^, resulting in readable photocurrents. Due to the feasibility of the plasmonic nanostructures, the direct tuning of the photoresponse in terms of the operation wavelength and polarization dependence can be realized^43,44^, offering new solutions for optical detection applications. Currently, the fixed-size nanostructures resonate only at specific wavelengths, which limits multi-wavelength applications, and the difficulty of processing metallic nanostructures rises progressively as the operating range broadens (Fig. 1b(iii))^45,46^.

In this work, we construct a versatile platform that releases the constraints on material anisotropy for realizing high-performance broadband polarization photodetection with high photoresponsivity, large anisotropic ratio, and high on/off ratio based on fully 2D materials and demonstrates its applications in IR optical communication and polarized artificial vision. Our method integrates MoS_2_-based field-effect transistor (FET) as amplification unit and WSe_2_-based photodetector (PD) as polarization-sensitive unit (Fig. 1c(i)). The polarization originates from hot electron injection caused by the plasmonic metal electrode. The combined amplification system (ASPD) improves the polarization ratio from around 2 of single WSe_2_-metal PD to over 60 in IR band, mitigating the dependence on the intrinsic anisotropy of the material while already reaching the practical application level^36,47^. The system also exhibits high on/off ratio, which are 4.4 × 10^3^ and 5.7 × 10^5^ in IR and visible band, respectively. Since the integrated system greatly improves detection capabilities, we utilize the constructed system to implement polarization-modulated IR optical communication, which demonstrates perfect reproduction of the input signal, as well as polarized optical imaging and artificial neural network-based image recognition with a high recognition accuracy ~99% (Fig. 1c(ii)). Our results exhibit large promise for use in secure optical communication^8^, hyperspectral imaging^48^, and artificial vision et al.^14^, providing a viable avenue for the development of high-performance multifunctional on-chip optoelectronic systems.

## Results

### Working principle and application schematics of the proposed system

**Fig. 2:**
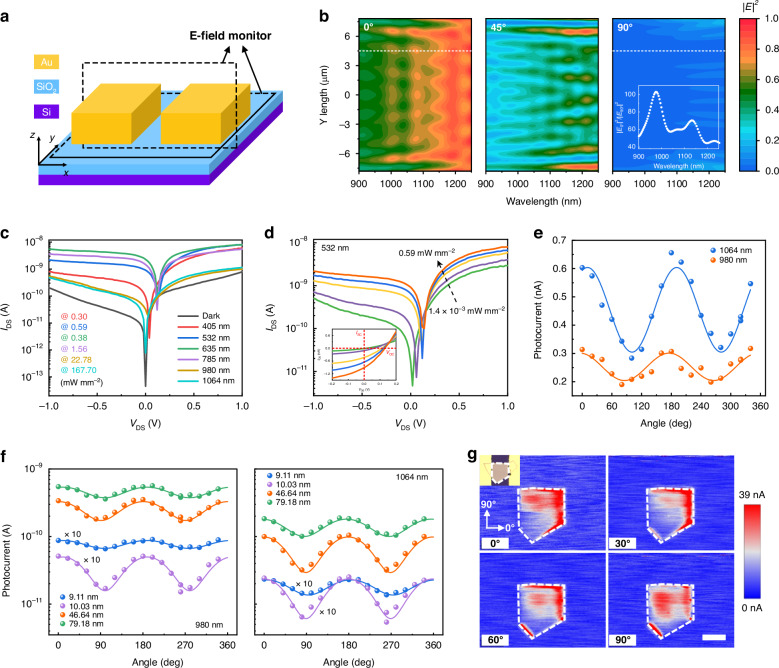
Design and characterization of the WSe_2_-based photodetector with polarization sensitivity. **a** Schematic of the simulated device model. **b** Spatial distribution of wavelength-dependent electric field at the edge of the device electrode at different polarization angles of 0° (along x direction shown in **a**), 45°, and 90° (along y direction) in the x-y plane. The inset plots the ratio of the field strengths in the two perpendicular directions versus the wavelength at the boundary extracted along the white dashed line. **c** Output curves of the WSe_2_ photodetector in the dark and under different incident light illuminations. *V*_DS_ denotes source-drain voltage and *I*_DS_ denotes the output current, respectively. **d** Output curves of the WSe_2_ photodetector under 532 nm illumination at varying incident powers. The inset is a detailed linear-scale magnification of the output curves, where *I*_sc_ and *V*_oc_ represent the short-circuit current and open-circuit voltage, respectively. **e** Photocurrent versus polarization angle of the WSe_2_ photodetector under *V*_DS_ = −1 V, illuminated by 980 nm light at 6.06 mW mm^−2^ and 1064 nm light at 10.66 mW mm^−2^. **f** Photocurrent versus polarization angle of WSe_2_-based photodetectors with different channel thicknesses. **g** Spatially resolved photocurrent mapping images at *V*_DS_ = −1 V for different polarization angles, with the mapping set perpendicular (0° polarization) and parallel (90° polarization) to the electrode edges. The left inset is the optical image of the device. The illumination wavelength for photocurrent mapping measurement is 1064 nm. The white lines outline the WSe_2_ channel. The scale bar is 10 μm

We first theoretically investigated a metallic contact interface on Si/SiO_2_ substrate, as shown in Fig. 2a. The theoretical design and modeling are performed using finite-difference time-domain (FDTD) simulation, with specific dimensions detailed in Supplementary Note 1. It is worth pointing out that hot electrons have very short lifetimes, in which the thermal relaxation process is completed in less than a few picoseconds. In this case, hot electrons should be collected or extracted (i.e., charge transfer) on the same time scale before they lose their excess energy. Therefore, we mainly focus on the electric field distribution at the edge of the electrodes, since the Schottky contact formed at the edge between the metal electrode and the semiconductor will be a good receptor for trapping hot electrons. Additionally, our work primarily focuses on the effect of electrode width, although the electrode length is also significant as it pertains to the actual contact length between the metal electrode and the channel material. To ensure the universality of our simulation results, we avoided specifying a fixed setting for the electrode length, thus not imposing a particular size limitation on the channel material. We simulated the electric field at the electrode edge (e.g., P point in Fig. S1a) along the two orthogonal polarization directions, parallel to and perpendicular to the edge, and found that there is obvious resonance peaks in IR band. The intensity of the peak increases as the electrode width decreases, and by reducing the dimensions of the electrode to sub-micrometer scales to match the plasmon frequency^37^, the sub-bandgap absorption increases with a concomitant blue-shift of the peak (Fig. S1b)^43^. Notably, as electrode width increases, the absorption finally stabilizes, which means the plasmonic metal electrode produces a plasmon resonance effect and will no longer be impacted by changes in electrode width.

The electric field strength at the electrode edge is polarization-dependent. To visualize this polarization-dependent electric field strength, the local electric field distribution at different wavelengths at the electrode edge for different polarization directions of 0° (along x), 45°, and 90° (along y) is shown in Fig. 2b. There is a significant difference in the spatial distribution at the three polarization angles, which reaches the largest when the incident light polarization angle is perpendicular to the edge (0° polarization) and lowest when parallel to (90° polarization), and intermediate between the two at 45° polarization. The electric field distributions at the electrode edge with different channel widths were investigated (Fig. S2a, b), and the field strength is significantly enhanced as the width decreases, which is consistent with the results in Fig. S1b. The spatial distribution of the field strength after increasing the channel length while keeping the same width shows that different lengths carry different degrees of field enhancement but still maintain the same polarization correlation and regularity (Fig. S2c). The injection yields of the hot carriers at the boundary follow the Fowler’s equation, considering the absorption of a photon produces only one hot electron-hole pair^49^:

1 $$\eta \sim {C}_{F}\cdot \frac{{(\hslash \nu -q{\phi }_{B})}^{2}}{\hslash \nu }$$

where *C*_*F*_ is a coefficient related to the Fermi energy in a given device, *ħν* is the incident photon energy, and *ϕ*_*B*_ is the Schottky barrier. Significantly, the injection yield *η* depends on the wavelength of the excitation light rather than the polarization, which does not explain the polarization dependence of the field strength distribution. In addition to this, we also need to take into account the absorption of the metal. The metal absorption (*I*_*abs*_) is proportional to the photo energy flux, which is related to its respective electric field through the time-averaged Poynting vector *S*^50,51^:

2 $${I}_{abs} \sim {\langle S\rangle }_{time} \sim {|E|}^{2}$$

Thus, we speculate that the polarization dependence observed in the electric field distribution is likely a consequence of the polarized light absorption by the plasmonic metal electrodes. This is theoretically in line with our anticipated design, specifically the introduction of polarization-dependent plasmonic metal electrodes leading to hot electron injection.

To support and validate our simulation results, we designed a PD with Schottky contact formed between plasmonic metal electrodes and 2D material, which serves as an electron capture for hot electrons. It is worth noting that we chose polarization non-sensitive WSe_2_ as the channel material to exclude the influence of polarization absorption of the channel area. The electrode width is fixed at 40 µm, which is the size where the resonance finally stabilized at IR band. Supplementary Note 2 provides details of the preparation process for the WSe_2_-based PD. Figure 2c plots the output curves (*I*_DS_–*V*_DS_) of the PD under dark conditions and light illumination from 405 nm to 1064 nm. From the curve signatures, we have come to two conclusions: first, the curves in dark shows the formation of Schottky contacts between the electrodes and the WSe_2_, and there is a significant asymmetric transport behavior, indicating a Schottky barrier difference exists at the contact interface between the source and drain electrodes with the WSe_2_. In general, interfacial electronic structure and density of state of 2D materials or interfacial trapped states are the main issues that induce the Schottky barrier^52^. Even with the same metal electrodes, asymmetric Schottky barrier, and built-in electric field may be caused^53^. Second, our device can work over a wide spectral range from the visible to the near-infrared (NIR), indicating that it has the potential to meet the needs of more application scenarios. We further analyzed the output current at different *V*_DS_ of the device under 532 nm illumination with varying power densities (Fig. 2d). The *I*_DS_-*V*_DS_ curve shifts upward as the power density increases from 1.4 × 10^−3^ to 0.59 mW mm^−2^, exhibiting a significant photovoltaic effect. The inset of Fig. 2d reflects the change of the short-circuit current (*I*_sc_) and open-circuit voltage (*V*_oc_) with the incident power densities. Similar performance was observed under other wavelengths of illumination (Fig. S4). We analyzed the device noise spectral densities (*S*_n_). At high-frequency region, the dark current in our device is the main source of noise. At low-frequency region, the *1*/*f* noise dominates the noise behavior of the device (Fig. S5 and Supplementary Note 3). The responsivity (*R*) at different wavelengths is extracted (Fig. S6)^53^. The PD has a maximum responsivity of 2.0 A W^−1^ and a detectivity of 1.1 × 10^11^ Jones at 532 nm. The detection performance in the NIR range is lower compared to the visible spectrum, a trend that aligns with the intrinsic absorption characteristics of the WSe_2_ (Fig. S7).

The device exhibits polarization-dependent photoresponse in the NIR range. Figure 2e demonstrates the photocurrent versus polarization angle at two typical NIR wavelengths, 980 nm, and 1064 nm. The anisotropic photocurrent ratios of the device are ~2 at 1064 nm and 1.5 at 980 nm, and both of them present the maximum photoresponse when the polarization direction is perpendicular to the edge of the electrode (0^o^ polarization), and the minimum when parallel to the edge (90^o^ polarization). Devices with different channel thicknesses were fabricated and showed a similar polarized photoresponse (Fig. 2f and details shown in Fig. S8). All devices are polarization-sensitive in the NIR range and are independent of the size and shape of the channel material. The spatially resolved photocurrent mapping images of a typical device (device in Fig. S9) show that the photoresponse is inhomogeneous and the photocurrent signal occurs mainly at the electrode edge (Fig. 2g). This phenomenon is consistent with our simulation results, where the hot electrons injected into the channel material through the Schottky interface leading to a polarization-dependent field enhancement at the electrode edge. The maximum photoresponse occurs when the direction of polarized light is perpendicular to the electrode edge (0° polarization), and the minimum value occurs when it is parallel to the edge (90° polarization).

The polarized photoresponse at visible wavelengths were also measured (Fig. S10), with no similar phenomenon in the visible range. The overall photocurrent of the device arises mainly from two mechanisms: one is the photocurrent generated based on the intrinsic absorption of the WSe_2_. And, the other is the hot electrons generated from the metal electrode. In the visible range, the photocurrent generated by the strong intrinsic absorption of WSe_2_ dominates. However, in the NIR range, the intrinsic absorption of WSe_2_ is very low (the absorption curves of WSe_2_ with different thicknesses are shown in Fig. S11), where hot electrons injection plays a major role. The polarization-sensitivity photoresponse is induced by the polarization-related field enhancement effect at the edge of metal electrode. Correspondingly, the electric field ratio (|*E*_0°_|^2^/|*E*_90°_|^2^) obtained from our simulation calculation has resonance peaks at NIR, which matches well with the phenomenon observed in our experiments, and the consistency between the two confirms our analysis. Notably, a low anisotropic ratio (~2) in our device is obtained, relative to the |*E*_0°_|^2^/|*E*_90°_|^2^ ^54–56^.

**Fig. 3:**
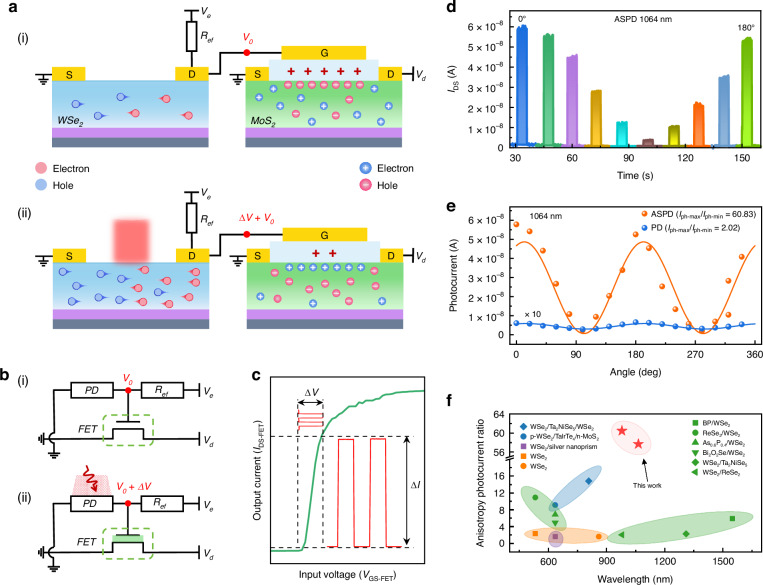
Polarization performance of the ASPD system. **a** Schematics of the ASPD system, depicting (i) the initial state in the dark and (ii) the working state under light illumination. R_ef_ denotes reference resistance. **b** Circuitry schematic of the ASPD system, illustrating (i) the initial state in dark; (ii) the working state under light illumination. In this schematic, PD denotes the WSe_2_-based photodetector, FET denotes the MoS_2_-based transistor. **c** Transfer characteristic of the integrated amplifier realized with MoS_2_ transistor, where ∆*V* represents the gate voltage variation induced by the input light signal, and ∆*I* denotes the corresponding output signal variation. **d** Time-resolved polarized photoresponse of the ASPD system under 1064 nm illumination at an intensity of 10.66 mW mm^−2^. **e** Photocurrent versus polarization angle for both the ASPD and the compared WSe_2_-based photodetector under 1064 nm illumination. **f** Comparison of the anisotropic ratio of the ASPD system against previously reported WSe_2_-based devices

It is noteworthy that the anisotropic ratio of PDs should be larger than 20 to ensure it is applicable in practical applications^36^. To achieve device performance improvements, a monolithically integrated amplification system for photodetection is constructed, in which a MoS_2_-based FET as an amplification unit is integrated with our WSe_2_-based PD. The schematic and optical image of the MoS_2_ FET is shown in Fig. S12, where hBN is used as the gate dielectric and graphene serving as the gate electrode. Figure 3a illustrates the structure of the integrated system and the generation process of photocarriers. Prior to the MoS_2_ FET being linked to the system, its fundamental electrical characteristics were assessed (Supplementary Note 4). The device exhibits n-type behavior with an impressive on/off current ratio about 10^6^ at *V*_DS_ = 0.5 V. The subthreshold swing (*SS*) of the FET was calculated as 117 mV dec^−1^ ^57^, and the device has a low threshold voltage (*V*_TH_) of −4 V, which is crucial for the FET as an amplification unit. The function of the MoS_2_ FET as an amplification unit is to rapidly convert the input signal of the gate voltage into a change in output current. The device can respond to the changing input signals quickly (Fig. S13). All the above performance parameters of the FET meet the requirements of our amplification system.

To optimize the detection performance, the MoS_2_-based FET in the ASPD system, which serves as an amplification unit and output port, is expected to toggle between the on and off states. To establish a low initial current in the ASPD system, the transistor is set to a negative gate voltage slightly below the threshold voltage for conduction. As the gate voltage varies, the transistor switches states; therefore, the initial voltage *V*_e_ of this system is less than zero. The ASPD system works as follows: Under dark conditions (Fig. 3a(i)), the intrinsic carrier concentration of WSe_2_ is relatively low, as a result, the resistance of the PD is in a high-resistance state. This leads to the formation of a large negative voltage (*V*_0_) on the gate terminal of the MoS_2_-based FET, and the transistor operates in an off state with a low output current (Fig. S12b). We define this as the initial state and the schematic of the corresponding circuit is shown in Fig. 3b(i). When incident light irradiates on the WSe_2_ PD (Fig. 3a(ii)), the photogenerated electron-hole pairs increase the carrier concentration, at this time, the resistance of the PD decreases, and the reference resistance *R*_ef_ remains in a high-resistance state, which causes a voltage difference Δ*V* (Δ*V* > 0) at the gate of the transistor as illustrated in Fig. 3b(ii). The change in gate voltage causes the transistor to switch to the on-state and outputs a larger photocurrent. We define this as the working state. Since the gate voltage of the FET has been regulated to operate in the vicinity of the threshold voltage, which is near the subthreshold swing region, a slight variation in the gate voltage (Δ*V*) will result in a large variation in the output current (Δ*I*), causing the system to transit from its initial state to its working state and the output current changes by orders of magnitude (Fig. 3c). When the incident light is removed, the system will return to its initial state.

Figure 3d shows the time-resolved polarized photoresponse of the ASPD system under 1064 nm illumination at *V*_d_ = 0.5 V, *V*_e_ = −5.5 V. With rotating the polarization angles of incident light, the maximum and minimum values of the corresponding output current (*I*_DS_) are 60.48 nA and 3.04 nA, respectively, and the photocurrent is extracted with a large anisotropic ratio of over 60. In comparison, the bare WSe_2_-based PD exhibit a 30-times lower anisotropic ratio, as shown in Fig. 3e. The ASPD system shows similar results under 980 nm illumination (Fig. S14). More detailed experimental results are summarized in Supplementary Table 1. It should be noted that the polarization performance of ASPD system is not observed in the visible band, which is consistent with our previous analysis (Fig. S15). Moreover, the photosensitivity obtained under the same NIR illumination are improved by several orders of magnitude, with a peak on/off ratio of 4.4 × 10^3^ (1064 nm) and 8.3 × 10^3^ (980 nm), details as shown in Fig. S16 and Supplementary Table 2. These are extremely impressive figures and will provide the imaging system with an extremely high on/off ratio, especially for imaging applications in complex environments. Notably, similar improvement was observed under the visible range (Fig. S17 and Supplementary Note 5). The anisotropic ratio of our ASPD system far surpass previous 2D materials-based PDs in IR band (Fig. 3f and Supplementary Table 3), reaching the level for practical applications. In addition, compared to most research on intrinsic anisotropy of 2D materials and anisotropic nanostructure integration, our method is more universally applicable since it overcomes the limitations imposed by material anisotropy and features a relatively simple manufacturing process. The above results indicate that our ASPD system has great potential for applications in optical communication and polarized optical imaging.

**Fig. 4:**
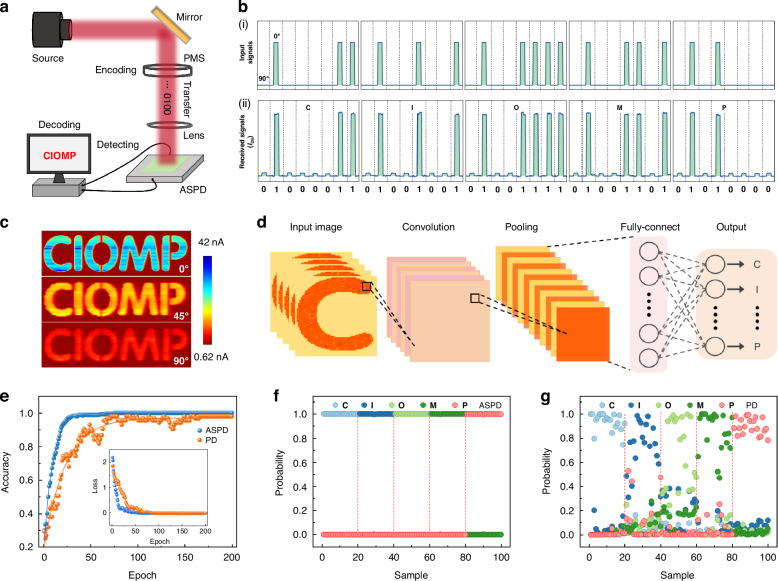
Proof-of-concept applications for infrared polarization communication and polarization imaging and recognition based on the ASPD system. **a** Schematic diagram of the experimental setup for a polarization-coded communication system. PMS: polarization modulation system. **b** (i) ASCII coding of “CIOMP” target encoded by the polarization angles (90° polarization is encoded as “0”, 0° polarization as “1”) of incident NIR light with a fixed power density. (ii) Received signals by our polarization amplification system and resolved the signal into the target “CIOMP”. The illumination wavelength is 1064 nm at an intensity of 10.66 mW mm^−2^. **c** Imaging results at distinct polarization angles: 0°, 45°, and 90°. **d** Schematic of the convolutional neural network (CNN) employed for image recognition. **e** Analysis of the recognition accuracy for polarization images captured by the ASPD system and a compared bare WSe_2_-based photodetector (PD). The inset shows the loss curves. **f**, **g** Probability of five possible results after inputting image samples of the ASPD and the compared bare WSe_2_-based photodetector (PD) into a trained neural network

To verify the practical utility of our system, which has ultrahigh polarization sensitivity, we next applied it for NIR optical communication and optical imaging. NIR optical communication delivers high security and immunity to electromagnetic interference. Integrating polarization detection with the communication system adds a new modulation dimension, further amplifying its application potential^58,59^. By modulating the polarization state of incident light (90° polarization is encoded as “0”, 0° polarization as “1”), the input information is converted into American Standard Code for Information Interchange (ASCII) code for information encoding, generating a binary data stream in NIR band carrying the “CIOMP” IR optical signal. The signal is then detected by our system, transmitted to the terminal computer, and complete the decoding process (Fig. 4a). Figure 4b(i) shows the input ASCII signal of the target “CIOMP” encoded by polarization angles of the incident light, and Fig. 4b(ii) shows the output current received on our ASPD system, obviously, the signal presents a perfect square wave, proving that our detection system can accurately identify polarization angle changes. Additionally, the strong correlation between the received signal and the original input information indicates that the system can transmit and reproduce information. This application demonstrates the implementation of an optical communication scheme with polarization regulation since the ASPD detection system we prepared is highly dependent on the polarization angle of incident light, which has great application potential in secure optical communications and related fields in the future^60^.

The high anisotropic ratio, excellent photosensitivity of our ASPD system can expand its functional diversity to distinguish the details of target objects, providing a feasible way for high-quality imaging^61–63^. Fig. S20 and Supplementary Note 6-1 show the real-time imaging process using the ASPD system. Figure 4c shows the photocurrent mapping images extracted by the ASPD system under 1064 nm light illumination with different polarized angles of 0°, 45°, and 90°. Distinct profiles of the “CIOMP” patterns are successfully produced under various polarization angles, demonstrating excellent NIR optoelectronic imaging capability, especially polarized imaging. With the rapid development of the application of artificial intelligence, neural network algorithms that simulate the brain can perform more efficient and faster information processing, such as image recognition, and they can be combined with image sensors to realize a simulation of human vision. Based on the optical imaging of our ASPD and bare WSe_2_ PD systems, we further introduced them into a convolutional neural network (CNN) for image recognition training and classification^64,65^. Supplementary Note 6-2 describes in detail the source of the image database and the construction process of the CNN, and the specific structure of the CNN is shown in Fig. 4d. Figure 4e shows the training accuracy of the two systems for comparison, the ASPD system achieves over 99% recognition accuracy in only about 25 training epochs, which is three times shorter than the epochs required for the PD system to achieve similar accuracy, which significantly increases convergence speed and reduces energy consumption. Under the same imaging conditions, the high photosensitivity of the ASPD system helps to minimize the impact of background noise on the imaging results, highlighting letter features more effectively and providing richer image details. To validate the CNN training results, we used our constructed test database (20 samples per letter and 100 samples per device) imported into two trained neural networks for recognition classification. The probability of generating five possible outcomes for each image was calculated (Supplementary Note 6-2). Figure 4f, g show the results of the two systems, respectively, where the scatter of each color indicates the recognition probability of the corresponding letter. Compared with the PD system, the ASPD system significantly improves the probability of predicting the correct result to a level close to perfect prediction, indicating that the ASPD system can markedly enhance the image recognition rate.

## Discussion

In conclusion, we have proposed and constructed an on-chip polarization detection system with high photoresponsivity, large anisotropy ratio, and on/off ratio based on fully 2D materials. We have demonstrated the WSe_2_ Schottky photodetector with polarization-sensitive properties by employing a strategic design of the metallic plasmonic contact interface, thereby eliminating the need for material anisotropy. By integrating MoS_2_-based FET as an amplification unit, we have significantly enhanced the anisotropic ratio of the WSe_2_-based PD from 2 to over 60. Moreover, this integrated platform has substantially improved the device on/off ratio by several orders of magnitude. Our method is straightforward and holds large potential for polarization photodetector applications, readily adaptable to various material platforms spanning isotropic to anisotropic materials. This adaptability broadens its applicability across a spectrum of scenarios. As demonstrations, we implied the developed system for polarization-modulated optical communication achieving signal reproduction. The system also delivers exceptional image recognition through artificial neural networks reaching an accuracy rate of over 99%. It should be noted that the Schottky barrier formed between the metal and 2D materials can be further modulated to facilitate the injection of hot electrons from the plasmonic metal, for instance by optimizing interface gap states and surface modification^66,67^. And, the plasmon resonance characteristics may be tuned in different plasmonic metals, including the intensity and bandwidth^10,68,69^. Our approach offers a new design paradigm for future polarization PDs, particularly for on-chip optoelectronic applications.

## Materials and methods

### Device fabrication

#### WSe_2_-based PD fabrication

WSe_2_ nanosheets were fabricated via the mechanical exfoliation method from bulk crystals (sixCabon Technology Shenzhen, China) using scotch tape and then transferred onto the SiO_2_/Si substrate (with 300 nm SiO_2_). Then, the devices were fabricated by ultraviolet photolithography and deposited Ti/Au (10 nm/80 nm) as electrodes using thermal evaporation.

#### MoS_2_-based FET fabrication

Firstly, the few-layer MoS_2_ was exfoliated from its bulk crystal using scotch tape. Then a micromanipulator (METATEST Corporation, China, E1-T) was used to transfer the MoS_2_ onto the SiO_2_/Si substrate (with 300 nm SiO_2_). Then, thin hBN was exfoliated onto the polydimethylsiloxane film and transferred on top of the MoS_2_ flake under the optical microscope assisted by an aligned transfer system, and the same method was utilized for the transfer of few-layer graphene onto the hBN. Next, to fabricate the device, the source/drain and gate electrode patterns were defined by ultraviolet photolithography, and Ti/Au (10 nm/80 nm) metals were deposited by thermal evaporation.

#### ASPD system fabrication

The drain terminal of the WSe_2_-based PD is connected to the gate of the MoS_2_-based FET. The reference resistor is designed to have a value close to the equivalent resistance of the PD under dark conditions at weak voltages for voltage dividing.

#### Characterization of the 2D nanosheets

The morphologies of the 2D nanosheets were investigated by an optical microscope (BX51, OLMPUS). The Raman, PL, and photocurrent mapping were carried out at room temperature by a confocal Raman/ PL system (Alpha 300 R, WITec) equipped with visible to IR laser sources. The thickness measurements of the flakes were carried out with atomic force microscopy (Cypher S, Asylum Research). The absorption spectra of the materials were collected by employing a customized microfocused absorption system.

#### Optoelectrical measurements

The electrical measurements were carried out at room temperature. All electronic and photoelectronic measurements were characterized by a semiconductor parameter analyzer (Keithley 4200) on a probe station (EVERBEING, C-4) under darkness and illumination at various light illuminations from visible to IR.

#### Polarization-sensitive characterization

A linear polarizer (Thorlabs, LPVIS050) and a half-wave plate were deployed to produce polarized light that illuminate on the sample, facilitating the measurement of polarization characteristics. Adjustments to the polarization angle were made by rotating the half-wave plate.

#### Photocurrent mapping

The spatial-resolved photocurrent mapping was conducted using scanning photocurrent microscopy built on a confocal Raman/PL system (WITec, Alpha 300 R). The focused laser beam (1064 nm) was raster-scanned over the whole device area, where the laser spot size is around 1.4 µm and the spatial resolution of the measurement nearly aligns with the laser spot size. The source-drain current was measured by a current meter under various polarization angles at *V*_DS_ = −1 V.

#### Noise spectral density

To obtain the noise current of the device, we performed the time-resolved dark current measurement on the device at a bias voltage of −1 V (Fig. S5a). The noise spectral density (*S*_n_) (Fig. S5b) was obtained by calculating the Fourier transform of the dark current. Detailed information is shown in Supplementary Note 3.

#### FDTD simulations

FDTD was used for the numerical simulations and calculated E-field spacial distribution at the edge of metal electrode. The 3D model was established according to the designed dimensions. A perfectly matched layer surrounds the entire simulation space, which was used to absorb any fields reaching the boundaries. The light source was defined as a plane wave. The detector monitors were placed to measure the distribution of the E-field. The optical constants of Au, SiO_2_, and Si were taken from Palik^70^.

## Supplementary information

Supplementary Information

## Data Availability

All technical details for producing the figures are enclosed in the supplementary information. All raw data are available from the corresponding authors upon request.
