# Supplementary material for: Versatile on-chip polarization-sensitive detection system for optical communication and artificial vision
Source: Light Sci Appl. 2025 Feb 3;14:68. doi: 10.1038/s41377-025-01744-x (PMC11790936; doi:10.1038/s41377-025-01744-x)
Supplement: Supplementary file 1 — Supplementary Information [file 41377_2025_1744_MOESM1_ESM.docx]

**Supplementary Information for**

**Versatile on-chip polarization-sensitive detection system for optical communication and artificial vision**

Zhilin Liu,^1,2^ Mingxiu Liu,^1,2^ Liujian Qi,^1^ Nan Zhang,^1^ Bin Wang,^1^ Xiaojuan Sun,^1^ Rongjun Zhang,^3^ Dabing Li,^1^ Shaojuan Li^1,*^

^1^ Key Laboratory of Luminescence Science and Technology, Chinese Academy of Sciences & State Key Laboratory of Luminescence Science and Applications, Changchun Institute of Optics, Fine Mechanics and Physics, Chinese Academy of Sciences, Changchun, Jilin 130033, China.

^2^ University of Chinese Academy of Sciences, Beijing 100049, China.

^3^ Department of Optical Science and Engineering, Shanghai Frontiers Science Research Base of Intelligent Optoelectronics and Proception, Institute of Optoelectronics, Fudan University, Shanghai 200433, China.

*Address correspondence to (S. Li) [lishaojuan@ciomp.ac.cn](mailto:lishaojuan@ciomp.ac.cn)

**Supplementary Note 1：**

We used three-dimensional finite-difference time-domain (FDTD) simulation for simulation and calculation with the following model specifications. We chose Si/SiO_2_ as the substrate for constructing an electrode model that is dimensionally inconsistent in the x- and y-axis directions, where the material is gold (80 nm). The electrode length is 16 µm (i.e., Y length=16 µm, which is an initial value set based on the experience of prepared devices), which mainly depends on the length of the direct contact between the metal electrode and the channel material, considering the absence of contact with the material in the actual device does not affect the device (**Fig. S1a**). Then, for comparative analysis the effect of the electrode width (i.e., X width) on the field strength distribution at the edge of the electrode, we selected a range of widths, specifically X takes values in range of 1 to 40 µm. To illustrate the relationship between electric field strength and polarization, we compared the spatial distribution of the electric field for various electrode sizes. **Fig. S2a, b** show detailed views of the variation of the electric field at the edge of the electrode for three different polarization orientations for the same length with different widths of the electrode, where the Y length is 16 µm and the X widths are 6 µm and 30 µm, respectively. Comparative analysis reveals significant differences in the electric field along the electrode edge under three distinct polarization orientations. Additionally, it is observed that a narrower X width corresponds to a higher field strength, while also maintaining crucial symmetry along the y-axis. This conclusion is consistent with that of **Fig. S1b**, although our focus has shifted from discussing field strength variations at a specific point to examining the entire electrode edge. Besides, **Fig. S2c** (where X=20 µm, Y=20 µm) is compared to **Fig. 2b** (where X=20 µm and Y=16 µm) to demonstrate the effect of increasing the channel length on the spatial distribution of field intensity while keeping the width constant. This comparison reveals that different lengths result in varying degrees of field enhancement while maintaining consistent polarization correlations and patterns. Throughout the simulation, the light source is defined as a plane wave, and the entire simulation space is surrounded by a perfectly matched layer (PML) that absorbs any field that reaches the boundary. We constructed a light field monitor located at the interface between the electrode and the underlying SiO_2_, where the width of the monitor is 1 µm wider than the electrode, to capture the light field within 1 µm from the edge of the electrode. Typically, the channel width exceeds 10 µm in our design, indicating that the gap between the two electrodes is significantly larger than the average mean free path of hot electrons. Since the source and drain electrodes are fabricated with the same metal, to minimize computational workload, we employ a single electrode model for the simulation.

**Supplementary Note 2:**

The layered WSe_2_ flake was fabricated via the mechanical exfoliation method from bulk crystals, and then transferred onto a SiO_2_/Si substrate (with 300 nm SiO_2_). Before device preparation, we performed a series of material characterization. The energy-dispersive X-ray spectroscopy (EDS) spectrum of the WSe_2_ is presented in **Fig. S3a**, and the result agrees well with the stoichiometric of WSe_2_. The Raman spectroscopy of WSe_2_ was performed, where two characteristic peaks E_2g_^1^ and A_1g_ were observed at 248.3 cm^−1^ and 256.6 cm^−1^ respectively,^1^ as shown in **Fig. S3b**. The photoluminescence spectrum of WSe_2_ is depicted in **Fig. S3c** in which two peaks located at 1.40 eV (884 nm) and 1.59 eV (776 nm) can be clearly seen in the spectrum,^2^ which correspond to indirect and direct transition of excitons in multilayer WSe_2_. The corresponding thickness of the flake is determined to be 20.58 nm using atomic force microscopy (AFM) in **Fig. S3d**. The source/drain electrodes were patterned by ultraviolet photolithography, and Ti/Au (10/80 nm) metals were deposited by the thermal evaporation.

**Supplementary Note 3:**

To obtain the noise current of the device, we performed the time-resolved dark current measurement on the device at a bias voltage of -1 V (**Fig. S5a**). The noise spectral density (*S*_n_) is obtained by calculating the Fourier transformation of the dark current (**Fig. S5b**).^3, 4, 5^ Theoretically, the noise current is defined as the root mean square of the random fluctuations in the dark current at a detection bandwidth, and consists of these components:^3^

$\begin{matrix} i_{noise}=[i_{shot}^{2}+i_{thermal}^{2}+i_{1/f}^{2}+i_{g-r}^{2}]^{1/2} \\ =[2eI_{dark}B+\frac{4kTB}{R_{0}}+i\left(f,B\right)_{1/f}^{2}+i\left(f,B\right)_{g-r}^{2}]^{1/2} \end{matrix}$ (1)

where *I_dark_* is the dark current, *e* is the elementary charge, *B* is the bandwidth, *k* is the Boltzmann constant, *T* is the absolute temperature, and *R*_0_ is the shunt resistance of the device. The *i*(*f,B*)*^2^_1/f_* and *i*(*f,B*)*^2^_g-r_* are frequency-dependent noise components, conventionally referred to as *1*/*f* noise and generation-recombination (*g-r*) noise, respectively. They are caused by random fluctuations in the localized electronic states of the non-uniform surface of the material and will play a major role at low frequencies.^5^ And the first two items *i^2^_shot_* and *i^2^_thermal_* are defined as white noise, independent of frequency.

As shown in **Fig. S5b**, the *S*_n_ as a function of the frequency (*f*) contains two regions. At high frequency region, white noise dominates the noise behavior since the spectrum is almost frequency-independent. Therefore, the noise current ($i_{noise}$) can be expressed as:

$i_{noise}=\sqrt{2eI_{dark}B+\frac{4kTB}{R_{0}}}$ (2)

where $\sqrt{2eI_{dark}B}$ is shot noise and $\sqrt{\frac{4kTB}{R_{0}}}$ is thermal noise. Whereas *R*_0_ is derived to be ~2.1 GΩ from an *I*-*V* fitting at weak voltages in our device (**Fig. S4**), thus the $i_{thermal}^{2}={4kTB}/{R_{0}}$ is very small and even negligible compared to the shot noise.^6^ Therefore, the noise current can be estimated as $i_{noise}=\sqrt{2eI_{dark}B}$, indicating that the dark current in our device is the main source of noise. So, we can use the formula $D^{*}={{R\sqrt{A}}/\left( 2eI_{dark} \right)}^{1/2}$ to simplify characterizing the detectivity of the device, where *R* is responsivity and *A* is device active area. The calculated *D*^*^ of the device in the main text is 1.1×10^11^ Jones at 532 nm with a power density of 1.4×10^-3^ mW mm^-2^ at *V*_DS_=-1 V.

However, at low frequency region, the *1*/*f* noise dominates the noise behavior of the device. Thus, the specific detectivity can be expressed as: $D^{*}={R\sqrt{A}}/{S_{n}}$. According to this, the *D*^*^ is 1.63×10^11^ Jones at 532 nm for the device with a power density of 1.4×10^-3^ mW mm^-2^ at 1 Hz, which is very close to the detectivity (1.1×10^11^ Jones) calculated assuming the dark current dominants the noise current. To overcome or reduce dark current noise, the following measures can be taken: improving the preparation process to reduce defects and impurities in the material; using surface and interface treatment techniques to decrease defective states and dangling bonds; and controlling the operating temperature to lower the detector's operating temperature, thereby reducing thermally excited carriers and lowering the dark current.

**Supplementary Note 4:**

The schematic of the MoS_2_ FET is shown in **Fig. S12a**, and the transfer characteristics are displayed in **Fig. S12b** (the inset is a top-view optical image). The source-drain current (*I*_DS_) of the FET increases as the gate voltage (*V*_GS_) varies, indicating that it exhibits n-type behavior with an impressive on/off current ratio about 10^6^ at *V*_DS_=0.5 V. The subthreshold swing (*SS*) of the FET was calculated as 117 mV dec^-1^ according to:^7^

$SS=dV_{GS}/d[\log(I_{DS})]$ (3)

It has a low threshold voltage (*V*_TH_) of -4 V. To estimate the contact quality, the output characteristics of the FET at various *V*_GS_ are shown in **Fig. S12c**, the *I*_DS_-*V*_DS_ curves fluctuate almost linearly, suggesting that the electrodes and MoS_2_ have a near-ohmic contact. To illustrate the switching response of the transistor, we applied a square wave voltage (-6 V to 0 V) to the gate of the transistor at a frequency of 20 Hz under the condition of *V*_DS_=1 V (**Fig. S13a**), which illustrates that the transistor exhibits a good frequency response. Also, to estimate the switching speed of the FET, we extracted its response time and found that both the rise time and the fall time are less than 1.5 ms (**Fig. S13b**), which indicates that it can respond to the changing signals quickly. Meanwhile, both the on-state current (*I*_on_) and the off-state current (*I*_off_) were able to maintain the corresponding stabilized outputs over a long time range of 1000 s (**Fig. S13c**).

**Supplementary Note 5:**

While achieving anisotropic current ratio amplification, we observe that the ASPD system achieves equal amplification for other detection properties. **Fig. S17a-c** show the dynamic photoresponse curves of the APSD system and single WSe_2_-based photodetector (PD) under 532 nm, 635 nm, 785 nm illumination at *V*_d_=0.5 V, *V*_e_=-5.5 V. And the on/off ratio (*I*_light_/*I*_dark_) of the ASPD system is much higher than that of PD, the *I*_light_/*I*_dark_ of the ASPD can reach a maximum of 5.7×10^5^ under 532 nm at 14.73 mW mm^-2^. To clearly evaluate the on/off ratio under broadband spectrum, the response curves of the designed ASPD and PD under different wavelengths are depicted in **Fig. S17d**. Obviously, the on/off ratio of the ASPD was much larger, by a factor of 3.8×10^5^ (532 nm), than that of the PD at each wavelength evaluated in range from 532 to 1064 nm. In addition, there is no significant change in the photocurrent of the same intensity under different intensities of periodic light illumination, indicating good repeatability and stability of the response of the amplification system (**Fig. S18**). Furthermore, we presents the variation of responsivity with power under 532 nm light irradiation, as shown in **Fig. S19a**, the responsivity of ASPD can reach a maximum of 279.8 A W^-1^, which is two orders of magnitude higher than that of PD. We also evaluated the performance of the two systems at different wavelengths (**Fig. S19b**). It is evident that the ASPD exhibits significantly higher responsivity compared to the PD across the entire broadband spectrum. This further demonstrates that the amplification system is capable of providing equivalent amplification for multiple performance metrics of the photodetector.

**Supplementary Note 6-1:**

To further illustrate the imaging sensing capability of our system, the device is used as a single-point sensing pixel in imaging. **Fig. S20** illustrates a schematic diagram of the polarization imaging system. Within the system, a mask with hollowed-out letters “C, I, O, M, P” is placed in the path of the incident light, serving as the imaging target. A polarizer and a half-wave plate are utilized to modulate the polarization angle of linearly polarized light. During the imaging process, the mask is precisely moved stepwise in both horizontal and vertical directions by a computer-controlled two-dimensional motion platform. The incident light passes through the hollow parts of the mask and illuminates the detection system. A meter monitors and records the position-resolved photocurrent in real time. Then, a computer synthesizes the photocurrent and positional data into a mapped image. **Fig. 4c** displays the imaging results at 1064 nm, measured at polarization angles of 0°, 45°, and 90°, respectively, achieving a high-contrast image of the letters “CIOMP”.

**Supplementary Note 6-2:**

We have constructed two image databases to store the polarization images obtained by the ASPD and PD systems, respectively, with the images being acquired through real-time imaging tests under 0° polarization, where the images contain five letters, C, I, O, M, and P (**Fig. 4c** and **Fig. S21**). The resolution of each image obtained from the experimental tests was 512×512 pixels. For image recognition, we imported these two databases into the Convolutional Neural Network for recognition training. Each letter image has 100 samples in the database. We randomly divided the dataset into a training set and a test set, where 80% of the data was used for model training, and 20% was used for testing to ensure that the model could learn the generalized features. In our CNN model, the input layer of the network initially receives data from the image database, which has been processed to resize all images to 128×128 pixels uniformly. Subsequently, the convolutional layer employs the 3×3 convolutional kernel to extract image features. To enhance the stability and speed of training, we introduce Batch Normalization technology following the convolutional layer. The Rectified Linear Unit (ReLU) activation function is then applied at the activation layer, enabling the network to handle nonlinear problems and thus capture more complex image features. Through the process of max pooling, the pooling layer uses the 2×2 pooling window with a stride of 2 to reduce the spatial dimensions of the features while preserving critical information. The fully connected layer, located at the end of the network, maps the extracted features to the output space, with the output dimension set to 5, corresponding to the five predicted categories. Finally, the output layer utilizes the Softmax activation function to achieve the final classification output. This function transforms the raw outputs of the preceding layer into probabilities ranging from 0 to 1, with the sum equaling 1. These probabilities represent the likelihood of each class, facilitating multi-class classification. The class with the highest probability in the output distribution is identified as the predicted result for each sample, which is determined by comparing the predicted probabilities with the actual labels. During training, the learning rate is set to 0.05 and does not change. In order to enhance the robustness of the model, different levels of Gaussian noise with a standard deviation ranging from 0.01 to 0.2 were added to the training data, which allows the model to improve its adaptability.

**Fig. S1 Schematics of the simulation model. a** The electrode model. Point P, situated at the edge of electrode, is a selected test point within the x-y plane, representing the contact interface between the metal electrode and the substrate. **b** The variation in the field strength ratio at Point P with respect to wavelength along different polarization directions. 0^o^ polarization (along x direction shown in **a**) and 90^o^ polarization (along y direction).

**Fig. S2 Spatial distribution of electric field.** **a-b** The electric field distribution at the edge of electrode with X widths of 6 µm and 30 µm, respectively, for a fixed Y length of 16 µm. **c** The electric field distribution at the edge of electrode with both X and Y dimensions of 20 µm. In this configuration, 0^o^ polarization is perpendicular to the electrode edge, while 90^o^ polarization is parallel to it.

**Fig. S3 Characterization of the WSe_2_ flake. a** The energy-dispersive spectroscopy (EDS) of the WSe_2_ flake, which exhibits strong W and Se signals, in general agreement with the stoichiometric ratio of WSe_2_. **b** The non-polarized Raman spectra collected from the WSe_2_ flake. The excitation laser wavelength is 532 nm. **c** The photoluminescence spectra of the WSe_2_ flake. The A and I corresponds to the position of the exciton absorption peaks of WSe_2_. **d** Thickness characterization of WSe_2_ flake, the inset is the optical image of the device (the black scale bar is 20 µm).

**Fig. S4 Photoresponse at various wavelengths. a** Schematic of the WSe_2_-based photodetector. **b-f** The output curves (*I*_DS_-*V*_DS_) in dark and light illumination at different power densities under 405, 635, 785, 980, and 1064 nm, respectively. Panels **b-d** show a monotonic upward shift in the *I*_DS_-*V*_DS_ curves with increasing light power, indicating a distinct photovoltaic effect. Panels **e-f** exhibit a subtle upward shift in the curves as power increases, though the photovoltaic effect remains apparent. The insets provide a localized linear-scale magnification of the output curves.

**Fig. S5 Device noise. a** The time-resolved dark current at *V*_DS_=-1 V. **b** The noise spectral densities (*S*_n_) as a function of frequency at *V*_DS_=-1 V.

**Fig. S6 Device characterization under different illumination.** Responsivity under 532 nm, 785 nm, 980 nm, and 1064 nm at *V*_DS_=1 V. It is evident that the detection performance in the NIR range is significantly lower compared to the visible spectrum.

**Fig. S7 UV-vis-NIR absorption spectra of the WSe_2_ flake.** This figure displays the absorption spectra of a WSe_2_ flake with a thickness of 28.87 nm, the thickness closely approximating the device under evaluation (20.58 nm).

**Fig. S8 Polarized photoresponse of WSe_2_-based photodetectors with different channel thicknesses.** The insets provide optical images of the devices depicting the polarized light currents under irradiation at wavelengths of 980 nm and 1064 nm. The data clearly demonstrate a pronounced polarization response in all devices within the same production batch, with different material thickness or shape of the channel material.

**Fig. S9 Device Optoelectrical properties under various illumination. a** The output curves (*I*_DS_-*V*_DS_) in dark and under different illumination at 532, 635, and 785 nm. The dark *I*_DS_-*V*_DS_ curves reveal an asymmetric Schottky contact between the WSe_2_ and the channel material, exhibiting a rectification ratio exceeding 10^2^. **b** The output curves at 532 nm under varying incident powers demonstrate an upward shift of the *I*_DS_-*V*_DS_ curve with increasing power, indicating a distinct photovoltaic effect. **c** Time-dependent dynamic response curves at *V*_DS_=0 V under different incident light, showing the self-powered operation capability. **d** Time-dependent dynamic response curves at *V*_DS_=0 V under varying incident power densities. **e** The responsivity and detectivity of the device at 785 nm under varying incident powers at *V*_DS_=-2 V and 0 V. **f** The responsivity and detectivity of the device at *V*_DS_=-2 V and 0 V under different illumination at 532 (3.8×10^-4^ mW mm^-2^), 635 (1.1×10^-4^ mW mm^-2^), and 785 nm (5.7×10^-5^ mW mm^-2^).

**Fig. S10 Polarized photoresponse of the bare WSe_2_-based photodetector.** Plots of polarized photocurrents at different wavelengths at 532 nm (0.042 mW mm^-2^), 635 nm (0.060 mW mm^-2^) and 785 nm (0.51 mW mm^-2^) under *V*_DS_=-1 V. There is no significant polarized-sensitivity response of the device in the visible.

**Fig. S11 The absorption of WSe_2_ flakes with different channel thicknesses.**

**Fig. S12 Performance characterization of MoS_2_-based FET. a** The schematic of the transistor, where hBN is used as the gate dielectric and graphene serving as the gate electrode. **b** The transfer curves. The inset is an optical image of the device, the scale bar is 20 µm. **c** The output curves at various gate biases.

**Fig. S13 The switching performance of MoS_2_-based FET. a** Dynamic measurement of the FET during continuous cycling between on/off states (*V*_GS_=-6 V to 0 V) under *V*_DS_=1 V at 20 Hz. **b** The rise and fall time of the FET at 20 Hz. **c** Stability of the FET in open and close state.

**Fig. S14 Polarized photoresponse of the ASPD system.** Polarized photoresponse under 980 nm at power intensity of 6.06 mW mm^-2^. **a** The time-resolved polarized photoresponse. **b** The relationship between photocurrent and polarization angle. **c** The comparison of polarization performance of ASPD and bare WSe_2_-based photodetector (PD) respectively. The noise current varies slightly across different polarization angles, which can be attributed to system errors during rotating the polarizer. Notably, the noise current fluctuates randomly without exhibiting a periodic relationship with the polarization angle, suggesting no direct correlation exists between them. Furthermore, the variation in noise current is small compared to the polarization photocurrent, rendering its impact on photocurrent extraction negligible.

**Fig. S15 Polarized photoresponse of the ASPD system across the visible spectrum.** The polarized photoresponse of the ASPD system when irradiated with wavelengths at 532 nm (0.042mW mm^-2^), 635 nm (0.060mW mm^-2^) and 785 nm (0.51mW mm^-2^). The results indicate a lack of polarization sensitivity in the visible light range.

.

**Fig. S16 The dynamic photoresponse of the ASPD and bare WSe_2_-based photodetector (PD).** Time-resolved photoresponse measurements of the ASPD and PD under NIR-light illumination at 1064 nm with a light power intensity of 1065.73 mW mm^-^² and at 980 nm with an intensity of 528.40 mW mm^-^², respectively.

**Fig. S17 Dynamic response of the ASPD and the bare WSe_2_-based photodetector (PD).** Time-resolved photoresponse at *V*_d_=0.5 V, *V*_e_=-5.5 V under various wavelengths: **a** 532 nm at 0.88 mW mm^-2^, **b** 635 nm at 0.23 mW mm^-2^, **c** 785 nm at 1.54 mW mm^-2^. **d** Comparison of on/off ratio (*I*_light_/*I*_dark_) for the ASPD system and PD under different wavelengths. Inset is the enlarged part of *I*_light_/*I*_dark_ for the single WSe_2_-based photodetector.

**Fig. S18 Time-resolved photoresponse of the ASPD system.** The time-resolved photoresponse of the ASPD system under various incident power. These data demonstrate that the response changes with increasing light power, yet retains a consistent performance under steady irradiation conditions.

**Fig. S19 Performances of the ASPD System and WSe_2_-Based photodetector (PD). a** Responsivity at various incident powers at 532 nm. **b** Responsivity at different wavelengths, including 532 nm, 635 nm, 785 nm, 980 nm, and 1064 nm. It is worth noting that we observed similar performance enhancement effects under different powers or wavelengths.

**Fig. S20 a** Schematic diagram of the polarization imaging measurement system. **b** The hollow mask plate as the imaging target, The white scale bar in the image is 4 cm. During the imaging process, the mask is precisely and gradually moved in both horizontal and vertical directions by a computer-controlled two-dimensional motion platform. The step size for this movement is 500 µm, ensuring that the final sample size for each letter image is 512×512 pixels.

**Fig. S21 Polarization imaging with the bare WSe_2_-Based Photodetector (PD). The imaging results of the pattern “CIOMP” captured by the PD at 0° polarization angle. The results showcase the capability to resolve detailed images under specific polarization conditions.** The size of each letter is 512×512 pixels, where the schematic diagram of the polarization imaging measurement system with the imaging target and dimensions is shown in **Fig. S20**.

**Supplementary Table 1. Comparison of the polarized photoresponse performance of the ASPD and bare WSe_2_-based photodetector (PD).**

| **Device** | **Wavelength (nm)** | **Power density (mW mm^-2^)** | ***I*_py_ (A)** | ***I*_px_ (A)** | **Anisotropic**  **Ratio (*I*_py_/*I*_px_)** |
| --- | --- | --- | --- | --- | --- |
| **PD** | **980** | **6.06** | **3.04×10^-10^** | **2.03×10^-10^** | **1.50** |
|  | **1064** | **10.66** | **6.14×10^-10^** | **3.04×10^-10^** | **2.02** |
| **ASPD** | **980** | **6.06** | **3.93×10^-8^** | **6.81×10^-10^** | **57.71** |
|  | **1064** | **10.66** | **5.11×10^-8^** | **8.40×10^-10^** | **60.83** |

**Supplementary Table 2. Photosensitivity of the ASPD and bare WSe_2_-based photodetector (PD) under different light illumination.**

| **Device** | **Wavelength (nm)** | **Power density (mW mm^-2^)** | ***I*_light_ (A)** | ***I*_dark_ (A)** | **On/Off ratio**  **(*I*_light_ /*I*_dark_)** |
| --- | --- | --- | --- | --- | --- |
| PD | 532 | 14.73 | 4.97×10^-9^ | 3.34×10^-9^ | 1.49 |
|  | 635 | 0.49 | 5.01×10^-9^ | 3.71×10^-9^ | 1.35 |
|  | 785 | 1.56 | 4.94×10^-9^ | 3.61×10^-9^ | 1.36 |
|  | 980 | 528.40 | 3.62×10^-9^ | 3.06×10^-9^ | 1.18 |
|  | 1064 | 1065.73 | 3.66×10^-9^ | 3.24×10^-9^ | 1.12 |
| ASPD | 532 | 14.73 | 1.72×10^-8^ | 3.01×10^-14^ | 5.7×10^5^ |
|  | 635 | 0.49 | 2.01×10^-9^ | 1.94×10^-13^ | 1.0×10^4^ |
|  | 785 | 1.56 | 8.28×10^-9^ | 3.18×10^-13^ | 2.6×10^4^ |
|  | 980 | 528.40 | 8.81×10^-9^ | 1.05×10^-12^ | 8.3×10^3^ |
|  | 1064 | 1065.73 | 8.67×10^-9^ | 1.95×10^-12^ | 4.4×10^3^ |

**Supplementary Table 3. Comparison of the polarizaiton performance of WSe_2_- based ASPD systems with previous works.**

| **Material** | **Wavelength (nm)** | **Anisotropic Ratio (*I*_py_/*I*_px_)** | **Ref** |
| --- | --- | --- | --- |
| WSe_2_ | 532 | 2.3 | 8 |
| WSe_2_ | 860 | ~1.6 | 9 |
| WSe_2_/silver nanoprism | 635 | 1.6 | 10 |
| BP/WSe_2_ | 1550 | 5.88 | 11 |
| ReSe_2_/WSe_2_ | 532 | 10.9 | 12 |
| As_0.6_P_0.4_/WSe_2_ | 635 | 6.85 | 13 |
| Bi_2_O_2_Se/WSe_2_ | 635 | 4.9 | 14 |
| WSe_2_/Ta_2_NiSe_5_ | 1310 | 2.25 | 6 |
| WSe_2_/ReSe_2_ | 980 | 2 | 15 |
| WSe_2_/Ta_2_NiSe_5_/WSe_2_ | 808 | 14.8 | 16 |
| p-WSe_2_/TaIrTe_4_/n-MoS_2_ | 635 | 9.1 | 5 |
| **ASPD** | **980** | **57.71** | **this work** |
| **ASPD** | **1064** | **60.83** | **this work** |

**REFERENCES**

1. Dai, M. et al. High Performance Self-Driven Photodetectors Based on MoS_2_ Schottky Barrier Diode. *Advanced Optical Materials* **12**, 2301900 (2024).

2. Zheng, B.Y. et al. Color-Selective and CMOS-Compatible Photodetection Based on Aluminum Plasmonics. *Advanced Materials* **26**, 6318-6323 (2014).

3. Jansen-van Vuuren RD. et al. Organic Photodiodes: The Future of Full Color Detection and Image Sensing. *Advanced Materials* **28**, 4766-4802 (2016).

4. Huo, N. et al. MoS_2_–HgTe Quantum Dot Hybrid Photodetectors beyond 2 µm. *Advanced Materials* **29**, 1606576 (2017).

5. Han, X. et al. A Polarization-Sensitive Self-Powered Photodetector Based on a p-WSe_2_/TaIrTe_4_/n-MoS_2_ van der Waals Heterojunction. *ACS Applied Materials & Interfaces* **13**, 61544-61554 (2021).

6. Liu, M. et al. Photogating-assisted tunneling boosts the responsivity and speed of heterogeneous WSe_2_/Ta_2_NiSe_5_ photodetectors. *Nature Communications* **15**, 141 (2024).

7. Bie, Y-Q. et al. A MoTe_2_-based light-emitting diode and photodetector for silicon photonic integrated circuits. *Nature Nanotechnology* **12**, 1124-1129 (2017).

8. Xie, Z. et al. Ultimate Limit in Optoelectronic Performances of Monolayer WSe_2_ Sloping-Channel Transistors. *Nano Letters* **23**, 6664-6672 (2023).

9. Zhang, Y. et al. Optical Momentum Alignment Effect in WSe_2_ Phototransistor. *Advanced Optical Materials* **9**, 2002243 (2021).

10. Guskov A. et al. Polarization Sensitive Photodetectors Based on Two-Dimensional WSe_2_. *Nanomaterials* **12**, 1854 (2022).

11. Ye, L. et al. Highly polarization sensitive infrared photodetector based on black phosphorus-on-WSe_2_ photogate vertical heterostructure. *Nano Energy* **37**, 53-60 (2017).

12. Wu, W. et al. An Ultrasensitive ReSe_2_/WSe_2_ Heterojunction Photodetector Enabled by Gate Modulation and its Development in Polarization State Identification. *Advanced Optical Materials* **12**, 2301410 (2024).

13. Li, S. et al. Highly Sensitive Broadband Polarized Photodetector Based on the As_0.6_P_0.4_/WSe_2_ Heterostructure toward Imaging and Optical Communication Application. *ACS Applied Materials & Interfaces* **16**, 12805-12812 (2024).

14. Tao, L. et al. Raman Anisotropy and Polarization-Sensitive Photodetection in 2D Bi_2_O_2_Se–WSe_2_ Heterostructure. *ACS Omega* **6**, 34763-34770 (2021).

15. Ahn, J. et al. Near-Infrared Self-Powered Linearly Polarized Photodetection and Digital Incoherent Holography Using WSe_2_/ReSe_2_ van der Waals Heterostructure. *ACS Nano* **15**, 17917-17925 (2021).

16. Zheng, T. et al. Self-Powered Photodetector with High Efficiency and Polarization Sensitivity Enabled by WSe_2_/Ta_2_NiSe_5_/WSe_2_ van der Waals Dual Heterojunction. *ACS Applied Materials & Interfaces* **15**, 29363-29374 (2023).
